# Babies, Bottles, and Bisphenol A: The Story of a Scientist-Mother

**DOI:** 10.1371/journal.pbio.0050200

**Published:** 2007-07-17

**Authors:** Aimee Quitmeyer, Rebecca Roberts

## Abstract

A scientist and mother who studies bisphenol A, a chemical found in plastic baby bottles and cups, wrestles with the disconnect between scientific evidence that the chemical poses a special risk to children and current laws and regulations.

My 11-month-old daughter loves her baby bottles and sippy cups (first-person narrative is from the viewpoint of Rebecca Roberts). But as I sit and watch her drink from them, I cringe, because I happen to be a scientist who studies a chemical found in those bottles and cups. I also know that some scientific research suggests that exposure to that compound, called bisphenol A (BPA), is detrimental to good health—something I can't help but think about as I watch my daughter use her sippy cup as a teething ring.

As a scientist, I depend on evidence, logic, and imagination to explain observations made in the laboratory. I then interpret and communicate my findings to the scientific community and the public. As a mother, I strive to raise a healthy and happy child. I make daily decisions about what my baby does and does not do, in order to limit her exposure to danger. In both of my roles, I depend on information: nonbiased, factual, evidence-based information. The mother in me relies on my training as a scientist to objectively look at scientific data in order to determine personal choices for my daughter. But, like all people, I am not qualified, nor do I have the time, to understand all scientific issues. I must rely on others—on the brokers of information: other scientists, medical personnel, the government, regulatory agencies, corporations, nonprofit organizations, and the media, to name a few.

The purveyors of information are not necessarily as objective, however, in their interpretation and dissemination of scientific data as my scientific self would like them to be—influenced as they are by timing, money, convenience, politics, and countless other agendas. I can only hope that the “facts” I receive are objective. Moreover, I hope that any regulations stemming from this science are established for the benefit of my family and society as a whole. But how is the information regarding the effect of BPA on human health being packaged and communicated to the general public? Let's begin by understanding what BPA is and how our modern society relies on it.

In 1952, chemists working with BPA discovered that it could help form a hard, clear plastic called polycarbonate. Polycarbonates make such products as compact discs, sunglasses, bicycle helmets, water and milk bottles, baby bottles, food storage containers, tableware, plastic windows, bullet-resistant laminate, cell phones, car parts, toys, and some medical devices such as incubators, dialysis machines, and blood oxygenators. BPA is also used to make certain resins that are commonly found in the linings of food cans to prevent corrosion, and it is present in some polyvinyl chloride (PVC) plastic products, in white dental fillings, dental sealants, and in some flame retardants. In keeping with its widespread applications, BPA ranks among the highest-volume chemicals manufactured worldwide, with an annual production in 2003 of about 13 billion kilograms [1,2]. Regulation requiring a significant reduction in BPA production and use could have a dramatic economic impact and would likely require some changes in personal lifestyle.

BPA has been shown to leach from water bottles and food cans into the packaged foodstuffs. It then enters the body through the digestive tract when those foods are consumed. The level of BPA released from plastic depends on the age and wear of the plastic and on exposure to heat. For example, one study showed that small levels of BPA leached from baby bottles subjected to simulated normal uses, including boiling, washing with a bottle brush, and dishwashing [3]. Plastic tableware (such as those used in some schools) was also found to release BPA into hot vegetable soup [2]. Older, worn bottles and bowls released BPA more readily than newer products [2,3]. BPA is also present in rivers and streams and in drinking water, presumably due to leaching from plastic items in landfills [4–6]. A survey by the Centers for Disease Control and Prevention found that approximately 95% of Americans have detectable levels of BPA in their bodies [7].

Naturally, the prevalence of human exposure leads to questions about safety and health. Although the plastic industry continues to assert that BPA is safe, the chemical's endocrine-disrupting properties raise concern about its potential to cause harm. BPA exposure affects the hormonal system, in particular, the pathway involving estrogen; its effects have been studied on cells, tissues, and whole organisms. In adult male mice and rats, effects of BPA exposure—abnormal sperm and reduced fertility—were reversed when exposure stopped [8]. Of the few human epidemiological studies, one revealed a relationship between BPA exposure and repeated miscarriage [9]. Additionally, BPA causes a human breast cancer cell line to proliferate, indicating that estrogen-sensitive tissues and cells in the body may react similarly [10].

Many animal studies focus on the effect of BPA exposure during fetal development, when cells and tissues are especially susceptible to hormonal alterations. Not only does BPA disrupt proper functioning of the placenta during gestation, but it causes many deleterious health effects in offspring exposed in utero [11], including enlarged prostates, malformed urethra [12,13], and a higher risk of prostate cancer in male offspring [14], and genital tract alterations [12,13] and earlier puberty in female offspring [13]. Exposure also affects brain development, causing behavioral differences between males and females to be lost in offspring exposed in the uterus [15]. A similar correlation to human development is plausible. Indeed, BPA has been found in the bloodstream, placenta, cord blood, and fetal blood of humans at levels that are within the range studied in many of the animal models [16].

Although BPA was not used in plastics manufacturing until the 1950s, its hormonal activity was reported in 1936 [17]. For decades, products containing BPA were shown not to release the compound, and thus these products were deemed safe. Indeed, the current Environmental Protection Agency (EPA) regulation regarding allowable levels of BPA exposure is based on these early findings. As recently as 1999, an official of the Food and Drug Administration (FDA) stated that no BPA was detected in liquid stored in baby bottles under typical use conditions [18]. That same year, however, scientific techniques progressed such that very small levels of BPA could finally be measured accurately. Levels as low as parts per billion (ppb) are now routinely detected in the laboratory. Unfortunately, the ability to detect such low levels in a laboratory environment is often not good enough, since tissues and cells can respond to levels of BPA that are 100 times lower [19]. The first such study showing a detrimental effect of BPA at very low doses was published in 1997, and since then, over 100 other studies have been published [19,20].

Let's step back a moment and consider the roles of United States regulatory agencies such as the FDA and the EPA in determining the so-called safe human exposure level for a chemical. Founded in 1906 the FDA focuses on ensuring safety of food, drugs, and medical products. Much later, in 1970, the EPA was established to protect human health in general and safeguard the environment by consolidating the varied efforts of research, monitoring, standard-setting, and enforcement. Six years after the creation of the EPA, the Toxic Substances Control Act was passed by Congress. This Act gave the EPA the power to control chemicals that pose an unreasonable risk to human health or the environment. In other words, the EPA was charged with determining the safe human exposure level for chemicals. Since taking on this daunting task of monitoring the roughly 75,000 chemicals produced in or imported into the US, the EPA has taken action to reduce the risk of over 3,600 chemicals but has banned or limited the production or use of only five. Currently, the EPA lists the “safe level” for BPA as being 50 micrograms (or 0.00005 grams) of BPA per kilogram of body weight per day [21]. Following this guideline, a person weighing 140 pounds (approximately 63 kg) could “safely” ingest 0.003 grams of BPA per day, or a little over a gram of BPA each year. This “safe” level is much higher than the low doses to which people are routinely exposed.

At this point, you might be wondering why this is the first time you've ever heard of BPA. The information is out there but it is a puzzle to get through. Early studies indicated that BPA did not leach or leached in very small amounts from plastic products, including baby bottles. These studies are often referred to by those in the chemical industry, such as the American Plastics Council—who have a vested interest in maintaining the use of BPA in plastics production, to verify the safety of the products [22]. However, since 1999 many studies have shown that BPA leaches from products at levels known to cause health effects in animals. Earlier studies on BPA exposure also tended to find little resulting adverse health effects, yet these studies were often using doses that were higher than those now regarded as being in an environmentally relevant range—that is, the low doses that humans are exposed to regularly and that fit within the so-called “low-dose theory” that claims that lower doses can be more harmful than higher doses [23,24]. These were the main studies initially used by the EPA to determine the “safe” level of BPA exposure and that are often referenced to attest to the safety of BPA [21,22].

Because of this ambiguity, findings can be obscured by those who inform the public, especially those with a vested interest in BPA production and usage. As a result, the media presents a confusing and unclear picture of the health risks of BPA exposure by giving equal weight to statements from independent scientists and those working for industry. The resulting influence of this ambiguity was recently revealed in the spring of 2006 when US state legislators in California, Maryland, and Minnesota attempted to pass legislation that would ban the use of BPA in products aimed at children. None of them passed.

The bills focused on children because they are far more susceptible to adverse affects from chemical exposures than adults, even at very low doses. The biological processes involved in their ongoing development are vulnerable to disruption by BPA and their ability to metabolically detoxify such contaminants is not yet mature. Moreover, children are more likely to be exposed to BPA orally because of their need to put things in their mouth—a purpose for which some BPA-containing products, such as some baby bottles and teething rings, are specifically designed.

The California bill (AB319) was introduced in February 2005, making it the first such legislation to be introduced in any state. Sponsored by Assembly Member Wilma Chan (Democrat), AB319 called for any BPA-containing products, including toys or childcare articles, intended for use by a child 3 years old or younger to be prohibited in the state. (It also sought to ban other harmful chemicals such as phthalates.) Violators of the ban would face civil action, carried out by the Attorney General, and penalties of no less then US$10,000 for each day of violation [25]. The fact sheet accompanying the bill states, “AB319 recognizes that we must act now to prevent exposure by eliminating at the source the chemicals, such as Bisphenol-A and Phthalates that pollute our bodies. By making intelligent decisions about what chemicals we allow into the environment, we can prevent unnecessary exposures to dangerous substances. Furthermore, children are incredibly sensitive to chemical pollution…. Some chemicals are simply too toxic and dangerous to children, to allow exposures to continue.”

The bill was energetically opposed by stakeholders in the chemical, plastics, baby products, and grocery industries. Under the umbrella organization Coalition for Consumer Choice, the NoAB319 campaign successfully fought the bill both in the media and in the Assembly hearing. In a news release by NoAB319, Steve Hentges, executive director of the Polycarbonate Business Unit of the American Plastics Council, stated that the legislation was “founded on insubstantial claims and unproven hypotheses that lack scientific rigor.”

The contradictory information set forth by the proponents and opponents of the bill ultimately led to its death in the Appropriations Committee, even after an amendment removed the BPA provisions, because of one vote. San Francisco Democrat Leland Yee, according to a spokesman, “decided that the decisions to ban chemicals should be left to health experts, not politicians, especially after scientists gave conflicting testimony at an Assembly hearing last week.” [26]. Fortunately, Chan intends to resubmit the bill and Yee said he “would support a new bill if it authorized state health officials to evaluate the risks and make the decision.” [26].

At a more local level, the first legislation to ban BPA from products aimed at children passed in the city of San Francisco. The “Stop Toxic Toys” bill was virtually identical to AB319 and was signed into law on June 16, 2006. However, in April 2007, the clause limiting BPA in child-aimed products was repealed pending action at the state level. As a result, no action on BPA-containing products will occur in the city until January 2008, and only then if the state has not taken appropriate actions to reduce its use at the state level. While the initial San Francisco legislation was an important step, such a piecemeal approach to controlling BPA exposure, especially in young children, is not perfect. Companies and businesses are bound to have difficulty conforming to a variety of regulations. Although BPA-free alternatives are often available, consumers in areas with legislation may find a lack of choices when it comes to plastic products on the store shelves. Ideally the national regulatory agencies should step in to minimize these problems.

At the national level, the White House disputes the “low-dose theory” and has proposed funding cuts for EPA research on endocrine disrupting chemicals such as BPA; however, the US Congress has maintained the funding level [27]. The EPA has revisited safe exposure levels of other chemicals. For example, in 2001, the EPA reduced the allowable level of arsenic in drinking water from 50 ppb to 10 ppb. It should do the same for BPA.

Currently, both the EPA and the European Food Safety Authority (EFSA) have set the “safe” level of exposure to BPA to 0.00005 grams per kilogram of body weight per day. Although difficult to estimate accurately, humans are typically exposed to about 0.000001 grams of BPA per kilogram of body weight per day. This is 50 times lower than the EPA- and EFSA-deemed “safe” limit. Unfortunately, this level of exposure is still significantly higher than the low doses that some studies have shown to cause adverse health effects. Moreover, the levels of BPA found by the Center for Disease Control and Prevention to be present in the bodies of Americans appear to be too high to be explained by exposure to known sources of BPA [7]. Thus, there is a clear need for further health studies on BPA exposure and for regulatory agencies to continue to monitor the science behind the politics. An attentive assessment of the risk of human exposure to BPA may prompt the plastics industry and manufacturers of products containing BPA to reevaluate their use of BPA and opt for BPA-free alternatives.

In the meantime, what is the scientist-mother to do? The mother in me still waits anxiously for the regulatory agencies and the legislature to catch up with the research on BPA that the scientist in me appreciates. I have switched my brand of sippy cups to one that doesn't contain BPA (a quick internet search will yield many sites describing these and other BPA-free baby products). Nevertheless, while I feel proactive as I watch my daughter happily drink her water, I still cringe a little bit when she drops the sippy cup, toddles over to her toy bin, and starts to gnaw on her plastic turtle instead.

## Abbreviations

BPA: bisphenol A

## References

[pbio-0050200-b001] vom Saal FS, Richter CA, Ruhlen RR, Nagel SC, Timms BG (2005). The importance of appropriate controls, animal feed, and animal models in interpreting results from low-dose studies of bisphenol A. Birth Defects Res A Clin Mol Teratol.

[pbio-0050200-b002] Lyons G (2000). Bisphenol A: A known endocrine Disruptor.

[pbio-0050200-b003] Brede C, Fjeldal P, Skjevrak I, Herikstad H (2003). Increased migration levels of bisphenol A from polycarbonate baby bottles after dishwashing, boiling and brushing. Food Addit Contam.

[pbio-0050200-b004] Coors A, Jones PD, Giesy JP, Ratte HT (2003). Removal of estrogenic activity from municipal waste landfill leachate assessed with a bioassay based on reporter gene expression. Environ Sci Technol.

[pbio-0050200-b005] Kolpin DW, Furlong ET, Meyer MT, Thurman EM, Zaugg SD (2002). Pharmaceuticals, hormones, and other organic wastewater contaminants in U.S. streams. Environ Sci Technol.

[pbio-0050200-b006] Kuch HM, Ballschmiter K (2001). Determination of endocrine-disrupting phenolic compounds and estrogens in surface and drinking water by HRGC-(NCI)-MS in the picogram per liter range. Environ Sci Technol.

[pbio-0050200-b007] Calafat AM, Kuklenyik Z, Reidy JA, Caudill SP, Ekong J (2005). Urinary concentrations of bisphenol A and 4-nonylphenol in a human reference population. Environ Health Perspect.

[pbio-0050200-b008] Toyama Y, Suzuki-Toyota F, Maekawa M, Ito C, Toshimori K (2004). Adverse effects of bisphenol A to spermiogenesis in mice and rats. Arch Histol Cytol.

[pbio-0050200-b009] Sugiura-Ogasawara M, Ozaki Y, Sonta S, Makino T, Suzumori K (2005). Exposure to bisphenol A is associated with recurrent miscarriage. Hum Reprod.

[pbio-0050200-b010] Singleton DW, Feng Y, Chen Y, Busch SJ, Lee AV (2004). Bisphenol-A and estradiol exert novel gene regulation in human MCF-7 derived breast cancer cells. Mol Cell Endocrinol.

[pbio-0050200-b011] Lee CK, Kim SH, Moon DH, Kim JH, Son BC (2005). Effects of bisphenol A on the placental function and reproduction in rats. J Prev Med Pub Health.

[pbio-0050200-b012] Markey CM, Wadia PR, Rubin BS, Sonnenschein C, Soto AM (2005). Long-Term Effects of Fetal Exposure to Low Doses of the Xenoestrogen Bisphenol-A in the Female Mouse Genital Tract. Biol Reprod.

[pbio-0050200-b013] Nikaido Y, Yoshizawa K, Danbara N, Tsujita-Kyutoku M, Yuri T (2004). Effects of maternal xenoestrogen exposure on development of the reproductive tract and mammary gland in female CD-1 mouse offspring. Reprod Toxicol.

[pbio-0050200-b014] Ho S, Tang W, Belmonte de Frausto J, Prins G (2006). Developmental exposure to estradiol and bisphenol A increases susceptibility to prostate carcinogenesis and epigenetically regulates phosphodiesterase type 4 cariant 4. Cancer Research.

[pbio-0050200-b015] Rubin BS, Lenkowski JR, Schaeberle CM, Vandenberg LN, Ronshein PM (2006). Evidence of altered brain sexual differentiation in mice exposed perinatally to low, environmentally relevant levels of bisphenol A. Endocrinology.

[pbio-0050200-b016] Schonfelder G, Wittfoht W, Hopp H, Talsness CE, Paul M (2002). Parent bisphenol A accumulation in the human maternal-fetal-placental unit. Environ Health Perspect.

[pbio-0050200-b017] Lawson Dodds RA (1936). Synthetic oestrogenic agents without the phenanthrene nucleus. Nature.

[pbio-0050200-b018] (1999). Endocrine/Estrogen Letter.

[pbio-0050200-b019] Vom Saal FS, Welshons WV (2006). Large effects from small exposures. II. The importance of positive controls in low-dose research on bisphenol A. Environmental Research.

[pbio-0050200-b020] vom Saal FS, Timms BG, Montano MM, Palanza P, Thayer KA (1997). Prostate enlargement in mice due to fetal exposure to low doses of estradiol or diethylstilbestrol and opposite effects at high doses. Proc Natl Acad Sci U S A.

[pbio-0050200-b021] Agency USEP (1988). Integrated Risk Information System: Bisphenol A (CASRN 80-05-7).

[pbio-0050200-b022] Group BAGI (2006). Bisphenol A.

[pbio-0050200-b023] Myers JP (2007). Our Stolen Future.

[pbio-0050200-b024] Myers P, Hessler W (2007). Does ‘the dose make the poison?’. Environmental Health News.

[pbio-0050200-b025] (2005).

[pbio-0050200-b026] Cone M (2006). Ban on use of toxic materials in baby products founders.

[pbio-0050200-b027] Waldman P (2005). Toxic Traces: New questions about old chemicals - Levels of risk: Common industrial chemicals in tiny doses raise health issue - Advanced tests often detect subtle biological effects; Are standards too lax?.

